# Experiential Learning Through Editorial Practice: The *Panamacani* Initiative in Pharmacy Education

**DOI:** 10.3390/pharmacy14040095

**Published:** 2026-07-01

**Authors:** Gustavo A. Hernández-Fuentes, Iván Delgado-Enciso, Alejandra E. Hernández-Rangel, Jesús E. Castrejón-Antonio, Héctor R. Galván-Salazar, Janet Diaz-Martinez, Nibardo Cobian-Gutiérrez, José Guzmán-Esquivel, Alicia Olvera-Montejano, Carmen Meza-Robles, Alberto M. Ramírez-Montes, Fabian Rojas-Larios, Gabriel Ceja-Espíritu, Daniel A. Montes-Galindo

**Affiliations:** 1 Department of Molecular Medicine, School of Medicine, University of Colima, Colima 28040, Mexico; ghfuentes@ucol.mx (G.A.H.-F.); ivadelga@fiu.edu (I.D.-E.); alejjandra49@gmail.com (A.E.H.-R.); frojas@ucol.mx (F.R.-L.); gcejae11@ucol.mx (G.C.-E.); 2 Faculty of Chemical Sciences, University of Colima, Coquimatlan 28400, Mexico; 3 State Cancerology Institute of Colima, Health Services of the Mexican Social Security Institute for Welfare (IMSS-BIENESTAR), Colima 28085, Mexico; hector_rgs@hotmail.com (H.R.G.-S.); carmen.qfb@gmail.com (C.M.-R.); 4 Department of Dietetics and Nutrition, Robert Stempel College of Public Health and Social Work, Florida International University, Miami, FL 33199, USA; jdimarti@fiu.edu; 5 Facultad de Ciencias Biológicas y Agropecuarias (FCBA), Universidad de Colima, Colima–Manzanillo Highway Km. 40, La Estación, Tecomán 28930, Mexico; jcastrejon3@ucol.mx; 6 Research Center in a Minority Institution, Florida International University (FIU-RCMI), Miami, FL 33199, USA; 7 Campus Colima, Tecnológico Nacional de México, Villa de Álvarez 28976, Mexico; ncobian01@hotmail.com (N.C.-G.); alicia.olvera@colima.tecnm.mx (A.O.-M.); 8 Clinical Epidemiology Research Unit, Mexican Institute of Social Security, Colima 28984, Mexico; jose.esquivel@imss.gob.mx; 9 Independent Researcher, Colima 28000, Mexico

**Keywords:** science communication, pharmacy education, editorial publishing, higher education, peer review, student participation, knowledge dissemination, institutional case study

## Abstract

Science communication is increasingly recognized as a key component of higher education; however, its integration into disciplinary training remains limited, particularly in pharmaceutical sciences. This study analyzes the *Panamacani* initiative as an institutional science communication initiative embedded within pharmacy education, based on editorial practice within the Pharmaceutical Chemist Biologist program at the Universidad de Colima, Mexico. A descriptive institutional case study was conducted using data from the first five issues (2023–2025), comprising 58 published contributions. Institutional participation, authorship profiles, and audience engagement were evaluated through descriptive statistics and editorial records. Results showed that 70.7% of contributions originated from the host institution, while 29.3% involved external institutions, indicating progressive expansion. Undergraduate students accounted for 44.8% of authorship, with a gradual increase in participation from postgraduate students and researchers. External contributions reached up to 50% in one issue, suggesting increased visibility beyond the host institution. Article visibility totaled approximately 1500 views, with applied health topics receiving the highest level of audience attention. These findings suggest that editorial-based initiatives may provide opportunities for student participation in science communication, peer review, and knowledge dissemination. However, the present study did not directly evaluate educational outcomes or competency development. This model may represent a scalable framework for integrating science communication activities into pharmacy education while fostering public engagement with science.

## 1. Introduction

Science communication (SC) has become an essential component of contemporary research systems, extending beyond knowledge production to its dissemination and societal appropriation [1,2]. Universities are increasingly expected not only to generate scientific knowledge but also to ensure its accessibility and relevance to broader audiences, particularly in areas that directly affect public health, technology and the environment. In this context, science communication plays a critical role in promoting scientific literacy, informed decision-making and public trust [3,4,5].

SC refers to the processes through which scientific knowledge is translated, disseminated, and made accessible to diverse audiences, including healthcare professionals, patients, policymakers, and the general public [6]. In pharmacy education, communication has traditionally focused on patient counseling and professional interactions; however, contemporary pharmaceutical practice increasingly requires the ability to critically evaluate, communicate, and disseminate scientific evidence beyond clinical settings [7]. Despite growing interest in science communication in higher education, relatively few studies have examined editorial-based science communication initiatives embedded within pharmacy training programs, particularly those involving student participation in scientific writing, peer review, and public dissemination activities [8,9].

In Mexico, pharmacy education is primarily delivered through the *Químico Farmacéutico Biólogo* (QFB) degree, a comprehensive undergraduate professional program that integrates pharmaceutical sciences, clinical and laboratory sciences, public health, and research training [10,11]. Unlike the Doctor of Pharmacy (PharmD) model commonly used in the United States, the QFB curriculum emphasizes a broad scientific foundation combined with professional competencies in medication management, clinical laboratory practice, and health-related research [10,11]. Although scientific communication and critical appraisal of evidence are increasingly recognized as essential competencies for contemporary pharmacy professionals [9,12], opportunities for structured participation in scholarly publishing, peer review, and science dissemination remain limited within traditional pharmacy curricula. Consequently, there is a need to explore innovative educational approaches that integrate authentic science communication experiences into pharmacy training.

Although the importance of science communication is increasingly recognized, its integration of communication training within disciplinary education remains limited. In many academic programs, particularly in specialized fields, communication skills are not systematically incorporated into curricula, resulting in a gap between scientific knowledge production and its effective transfer to society [13,14]. This limitation is especially relevant in pharmaceutical and health sciences, where understanding the appropriate use of medications and health interventions has direct implications for public well-being [15,16,17].

The consequences of this gap are evident. Limited public understanding of scientific concepts contributes to misinformation, inappropriate use of medications and challenges in interpreting scientific evidence. At the same time, students in these disciplines often graduate with strong technical competencies but insufficient experience in communicating complex information to non-specialist audiences. Addressing this disconnect requires educational strategies that integrate communication practices directly into academic training [8,18,19].

Editorial-based approaches represent a promising solution. By involving students in writing, peer review and publication processes, these models provide experiential learning environments that foster critical thinking, synthesis and audience-oriented communication. Beyond skill development, such approaches also generate tangible outputs that contribute to science dissemination and public engagement [20,21,22,23].

Experiential learning theory provides a useful conceptual framework for understanding the educational potential of editorial activities. According to this perspective, learning occurs through cycles of experience, reflection, conceptualization, and application, allowing students to construct knowledge through active participation in authentic tasks [24]. Activities such as scientific writing, peer review, manuscript revision, and editorial decision-making incorporate several of these elements by engaging participants in real-world scholarly communication processes. Likewise, practice-based and work-integrated learning approaches emphasize the value of authentic professional experiences as part of disciplinary training [25,26]. Within pharmacy education, such experiences may complement traditional instruction by providing opportunities to develop scientific communication and knowledge dissemination skills [12,23].

Within this framework, the *Panamacani* initiative emerged as a pedagogical strategy within the *Químico Farmacéutico Biólogo* (in Spanish) program at the Universidad de Colima, Mexico. Initially conceived as a classroom activity to improve students’ scientific communication skills, the initiative evolved into a structured editorial platform that integrates contributions from undergraduate students, postgraduate participants and researchers from multiple institutions. This evolution reflects the educational orientation of the model and its potential influence beyond the classroom [8,21].

The present study aims to descriptively characterize the development of the *Panamacani* initiative as a science communication model embedded within pharmacy education. Specifically, it examines: (1) institutional participation and expansion; (2) authorship patterns according to academic level and gender; (3) temporal changes in contributor composition; and (4) audience engagement across thematic areas. Through a descriptive institutional case study based on the first five issues of the journal, this work seeks to provide descriptive evidence regarding the development, participation patterns, and communicative reach of an editorial-based science communication initiative in higher education. Although the initiative is discussed within an experiential learning framework, the present study does not directly assess student learning outcomes, competencies, or perceptions. Instead, it focuses on describing participation patterns, institutional expansion, and audience engagement as indicators of the initiative’s development.

## 2. Materials and Methods

### 2.1. Study Design

This study was conducted as a descriptive institutional case study aimed at analyzing the development, participation patterns, and communication reach of the *Panamacani* science communication initiative. The study focuses on documenting and characterizing the evolution of a single institutional experience rather than evaluating educational outcomes or testing specific hypotheses [27,28]. The study combines institutional document analysis with descriptive quantitative indicators derived from editorial records and digital engagement data. Data were collected for all issues published between January 2023 and December 2025, representing the complete editorial cycle available at the time of analysis. Rather than evaluating educational effectiveness or testing causal hypotheses, this study seeks to document and characterize the evolution of *Panamacani* as an institutional science communication initiative embedded within higher education.

The analytical framework was organized around four predefined research questions addressing institutional participation, authorship characteristics, temporal evolution of contributors, and thematic patterns of audience engagement. Because the study was based on editorial records and publication metrics, no direct measures of educational outcomes, student competencies, attitudes, or perceptions were collected.

### 2.2. Context and Data Sources

Data were collected from the first five issues of the *Panamacani* journal (2023–2025), comprising a total of 58 published contributions [29].

Sources of information included: (1) editorial records from the Open Journal Systems (OJS) platform, (2) author affiliation data, (3) manuscript metadata (issue number, authorship type), (4) institutional participation records and (5) article-level engagement metrics (number of views). All data were obtained from internal editorial databases and publicly accessible journal content.

### 2.3. Variables and Classification Criteria

The analysis focused on three main dimensions: (1) Institutional participation: Author affiliations were classified as internal (Universidad de Colima) or external (other national institutions). (2) Authorship type: Contributors were categorized into three groups based on academic status: undergraduate students, postgraduate students and researchers [30]. Undergraduate contributors corresponded to students enrolled in the Químico Farmacéutico Biólogo (QFB) undergraduate degree program. Postgraduate contributors included both master’s and doctoral students, while researchers comprised faculty members, clinicians, and professionals affiliated with academic or research institutions. The inclusion of postgraduate students and researchers reflects the progressive expansion of the initiative beyond its original undergraduate educational setting and was considered relevant for characterizing changes in authorship composition over time. (3) Temporal evolution: Contributions were analyzed across the first five issues to identify changes in participation patterns over time [31,32,33,34]. (4) Communication impact: Article visibility was assessed using the number of views as a proxy indicator of audience engagement [35,36].

For descriptive reporting purposes, each article was classified into a predefined thematic category according to operational criteria established by the editorial team. Contributions were grouped into one of five thematic categories based on their primary focus: (1) Applied Health, including topics related to disease prevention, treatment, nutrition, pharmacology, and public health; (2) Biomedical Sciences, including physiology, pathology, molecular biology, and biomedical research; (3) Natural Products, including medicinal plants, phytochemistry, ethnopharmacology, and bioactive compounds; (4) Basic Sciences, including chemistry, biochemistry, microbiology, physics, and laboratory sciences; and (5) Science and Society, including science communication, ethics, education, policy, and social aspects of science. Classification was conducted through editorial consensus based on the principal content and objectives of each article [6]. In cases where articles could reasonably fit more than one category, classification was based on the predominant thematic emphasis of the manuscript. The purpose of this classification was solely to facilitate descriptive reporting of thematic distribution and audience engagement patterns and should not be interpreted as a qualitative analytical procedure.

### 2.4. Data Analysis

A descriptive statistical analysis was performed. Absolute frequencies and percentages were calculated for categorical variables, including institutional distribution and authorship type [37]. Temporal trends were evaluated by comparing participation patterns across issues. Data visualization (bar charts, pie charts and trend graphs) was used to facilitate interpretation of patterns in authorship and institutional participation. Data analysis was performed using SPSS version 22 software (IBM Corp; Armonk, NY, USA) [38].

Authorship classification was based on the academic level of the first author, considered the primary contributor to the manuscript. The first author was considered the primary contributor for classification purposes, as this position typically reflects the main responsibility in manuscript development within academic publishing practices [39]. Internal contributions correspond to authors affiliated with the Universidad de Colima, while external contributions include all other institutions. Counts represent the number of published contributions per issue.

Article views were obtained from the journal’s digital platform and represent the number of times each article page was accessed. Although this metric does not distinguish unique users, it provides a consistent proxy for audience engagement across issues.

Given the descriptive and exploratory nature of the study, no inferential statistical analyses were performed. The analysis was intended to characterize participation patterns, editorial development, thematic distribution, and audience engagement rather than establish causal relationships or test specific hypotheses [40].

### 2.5. Editorial Process and Publication Workflow

The *Panamacani* journal follows a structured editorial workflow aligned with standard academic publishing practices. All submitted manuscripts undergo a double-blind peer review process, in which both authors and reviewers remain anonymous.

Each manuscript is evaluated by at least two reviewers, who assess both scientific accuracy and communicative clarity. Based on these evaluations, editorial decisions are categorized as acceptance, minor revisions, major revisions, or rejection. Revised manuscripts are resubmitted and reassessed until a final decision is reached. This iterative process ensures both the quality of the scientific content and its accessibility to non-specialist audiences.

The journal maintains a semiannual publication frequency, with each issue including approximately 10 to 15 manuscripts. Accepted articles undergo professional editorial processes, including copyediting, layout design, and final proofing prior to publication. During the study period, approximately 62–64 manuscripts were submitted across the five publication cycles, of which 58 were ultimately published following peer review and revision. Manuscripts that met minimum quality standards but could not be accommodated within a specific issue due to space limitations were deferred to subsequent publication cycles rather than rejected.

Consequently, publication totals reflect both manuscripts submitted and manuscripts deferred from previous cycles. For this reason, cumulative submission and publication figures should not be interpreted as direct indicators of the rejection rate.

The implementation of a formal peer review system within a pedagogical framework distinguishes the initiative from purely instructional activities, positioning it as a hybrid model that integrates academic rigor with training objectives [41,42,43].

### 2.6. Ethical Considerations

The study is based on aggregated editorial data and does not involve human subjects or sensitive personal information; therefore, formal ethical approval was not required. All data were anonymized and aggregated for analytical purposes. As the study does not involve human subjects or experimental procedures, formal ethical approval was not required. For the editorial process *Panamacani* includes the following sentence in each publication: *Panamacani* is an institutional science communication journal published by the Faculty of Chemical Sciences at the Universidad de Colima. *Panamacani* operates under a Creative Commons Attribution-NonCommercial-ShareAlike 4.0 International License (CC BY-NC-SA 4.0). The content of published manuscripts is the sole responsibility of the authors and does not necessarily reflect the views of the journal or the Universidad de Colima [29].

## 3. Results

### 3.1. Institutional Participation and Expansion

**Figure 1:**
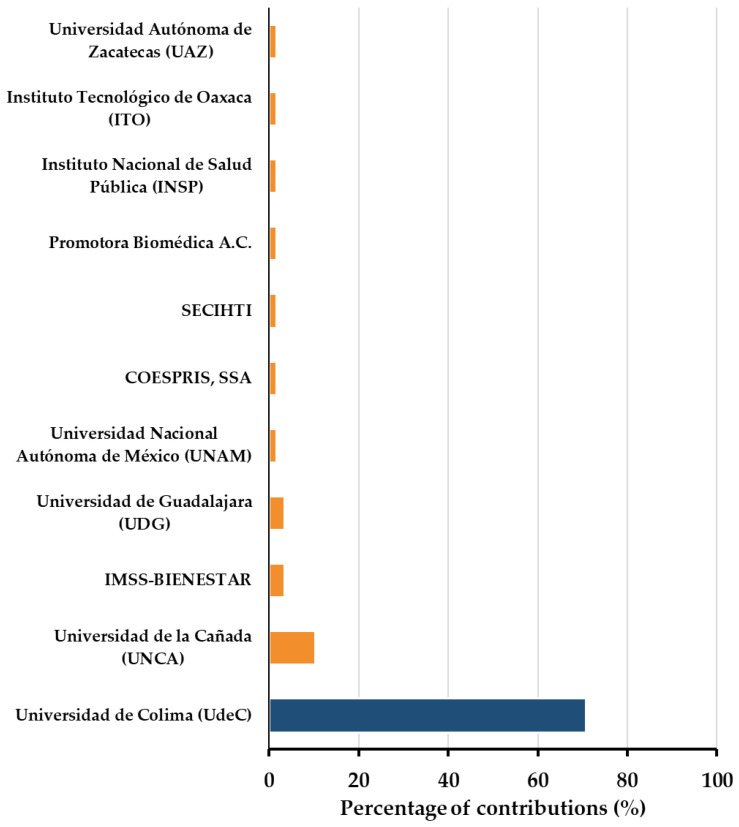
Distribution of contributions by institutional affiliation, showing the predominance of internal contributions and the emergence of external participation. The affiliations represented are: University of Colima (UdeC), University of La Cañada (UNCA), IMSS-BIENESTAR, University of Guadalajara (UDG), National Autonomous University of Mexico (UNAM), COESPRIS–Ministry of Health, Ministry of Science, Humanities, Technology and Innovation (SECIHTI), Promotora Biomédica A.C., National Institute of Public Health (INSP), Technological Institute of Oaxaca (ITO), and Autonomous University of Zacatecas (UAZ). Blue color indicates internal institutional affiliation, and orange color indicates external institutional affiliation.

A total of 58 contributions were analyzed across the first five issues of *Panamacani*. The majority of contributions originated from the Universidad de Colima (*n* = 41; 70.7%), while external institutions accounted for 29.3% (*n* = 17) (Figure 1).

Regarding the editorial process, approximately 62–64 manuscripts were submitted during the study period, of which 58 were ultimately published following peer review and revision. Some manuscripts that met editorial quality standards were deferred to subsequent publication cycles due to issue capacity constraints. Consequently, cumulative submission and publication figures should be interpreted within the context of the journal’s editorial workflow, which includes both immediate publication and deferred publication mechanisms.

Although *Panamacani* operates within a pedagogical framework, the editorial process maintains standards of scientific rigor. All submissions are subject to peer review, plagiarism screening and evaluation of citation practices, including the appropriate use of self-citations. The process emphasizes iterative feedback and revision as part of student training; however, manuscripts that fail to meet minimum academic, ethical or quality standards are not accepted.

This distribution indicates that, while the initiative remains institutionally anchored, it has progressively expanded beyond its original academic context. The participation of diverse institutions suggests increasing visibility and recognition as a science communication platform.

### 3.2. Authorship Profile and Educational Dimension

**Figure 2:**
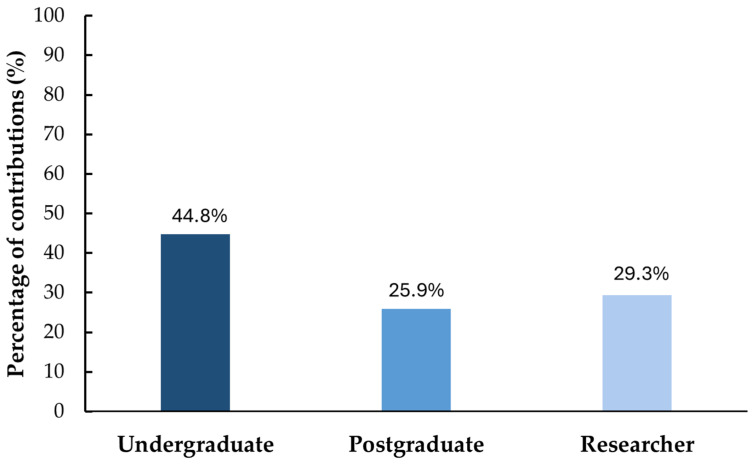
Distribution of authorship by academic level, illustrating the coexistence of undergraduate, postgraduate and researcher contributions. Authorship classification was based on the academic level of the first author, considered the primary contributor to the manuscript. Internal contributions correspond to authors affiliated with the Universidad de Colima, while external contributions include all other institutions. Counts represent the number of published contributions per issue.

Authorship analysis revealed a heterogeneous distribution of contributors. Undergraduate students accounted for 44.8% (*n* = 26), followed by researchers (29.3%; *n* = 17) and postgraduate students (25.9%; *n* = 15) (Figure 2).

This overall distribution highlights the dual nature of the initiative as both an educational initiative and a science communication platform. While undergraduate participation remains substantial, the involvement of postgraduate students and researchers reflects a diversification of expertise and increasing academic maturity within the publication.

When analyzed across issues, authorship patterns show a clear evolution over time (Table 1). In the first issue, undergraduate students represented most contributors (88.9%; *n* = 8/9). However, this proportion decreased in subsequent issues, reaching 20.0% in Issue 5 (*n* = 3/15). In contrast, postgraduate participation increased markedly, from 0% in Issue 1 to 66.7% in Issue 5 (*n* = 10/15), becoming the dominant contributor group in the most recent issue. Researcher participation remained relatively stable, with moderate fluctuations across issues. These findings indicate increasing participation from postgraduate students and researchers in later issues, resulting in a more diverse authorship composition.

An analysis across the five issues shows a shift in participation patterns over time. In the first issue, contributions were predominantly internal (*n* = 8) with minimal external participation (*n* = 1). By the fourth issue, internal and external contributions reached parity (*n* = 6 each), indicating increased external engagement. Although the fifth issue shows a reduction in external contributions (*n* = 3), the overall pattern suggests a progressive incorporation of external institutions into the journal (Table 2).

A similar trend is observed in authorship type. Early issues were dominated by undergraduate students (Issue 1: *n* = 8), whereas later issues show increased participation of postgraduate students and researchers. Notably, in Issue 5, postgraduate contributions (*n* = 10) exceeded undergraduate contributions (*n* = 3), reflecting a shift toward greater academic diversification (Table 2). Researcher participation was highest in Issue 2 (*n* = 6), remained moderate in Issues 3 and 4 (*n* = 3 and *n* = 4, respectively), and decreased in Issue 5 (*n* = 2). Overall, these patterns indicate an evolution from a predominantly undergraduate-driven initiative toward a more academically diverse authorship structure, consistent with the progressive consolidation of the journal.

### 3.3. Gender Distribution of Authorship

**Figure 3:**
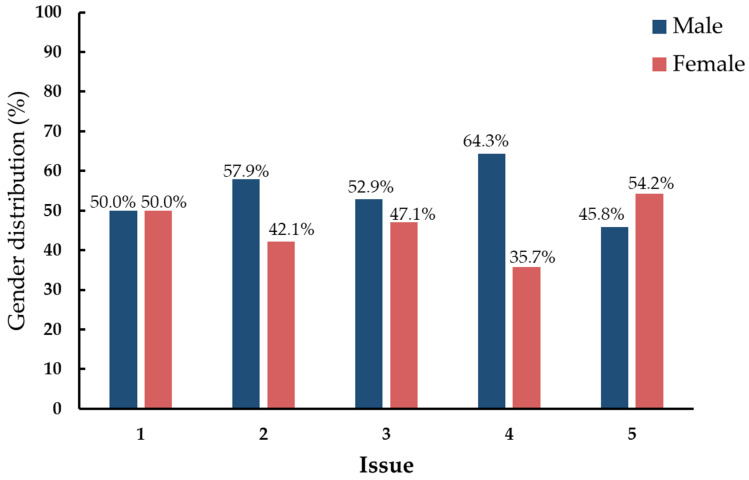
Temporal distribution of gender among first authors across the first five issues. Gender distribution based on first authors, showing variability across issues and a shift toward increased female participation in recent editions.

The analysis of authorship by gender revealed a relatively balanced distribution, with male authors accounting for 53.5% (*n* = 46) and female authors for 46.5% (*n* = 40). When examined across issues (Figure 3), gender participation shows variability over time. Early issues exhibit parity (Issue 1: 50% male, 50% female), followed by a predominance of male authors in Issues 2 and 4. In contrast, Issue 5 shows a higher proportion of female authors (54.2%), indicating a shift toward greater female participation. These patterns suggest a dynamic but overall balanced gender representation, consistent with trends observed in health and chemical sciences.

### 3.4. Communication Impact and Audience Engagement

Article-level engagement, measured by number of views, revealed variability in audience interest across topics. During the analyzed period, the journal accumulated a total of 1500 views, 585 sessions, and 410 new users, indicating sustained interaction and the capacity to attract new audiences.

The most viewed article, “El poder del magnesio: tipos y aplicaciones esenciales en salud”, reached 153 views, significantly higher than other contributions. Other highly viewed articles addressed topics such as neurophysiology of consciousness (54 views), sunscreen and aging (51 views), and medicinal plants for diabetes (57 views).

Overall, the most accessed articles tend to focus on practical health-related topics, including nutrition, pharmacology and disease management. In contrast, more specialized or abstract topics showed lower levels of engagement.

**Figure 4:**
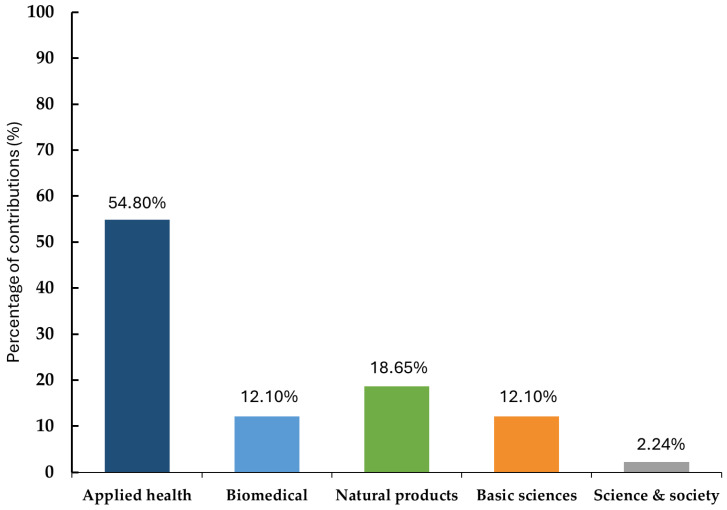
Distribution of views across thematic categories based on the ten most viewed articles. A total of 445 views were classified, showing a predominance of applied health-related content, followed by natural products, biomedical sciences, basic sciences and science and society topics.

These patterns indicate that applied health-related articles received more views than other thematic categories. When grouped by thematic area (Figure 4), audience engagement reveals a clear preference for applied health-related content. Considering the ten most viewed articles, a total of 445 views were classified into thematic categories. Applied health-related topics accumulated the highest number of views (*n* = 244; 54.80%), followed by natural products (*n* = 83; 18.65%), biomedical sciences and basic sciences (each *n* = 54; 12.10%), while science and society topics showed the lowest level of engagement (*n* = 10; 2.24%). However, article views should be interpreted as indicators of content visibility rather than direct measures of audience preference or impact.

### 3.5. Thematic Diversity and International Reach

The analysis of published content reveals a broad thematic diversity across the 58 contributions included in the first five issues of *Panamacani*. Articles are primarily distributed between chemical sciences and health sciences, reflecting the interdisciplinary scope of the journal. While chemical sciences predominate in early issues, later editions show an increasing presence of health-related topics, particularly those associated with clinical relevance and public health. Later issues included a greater proportion of health-related topics compared with earlier issues.

**Figure 5:**
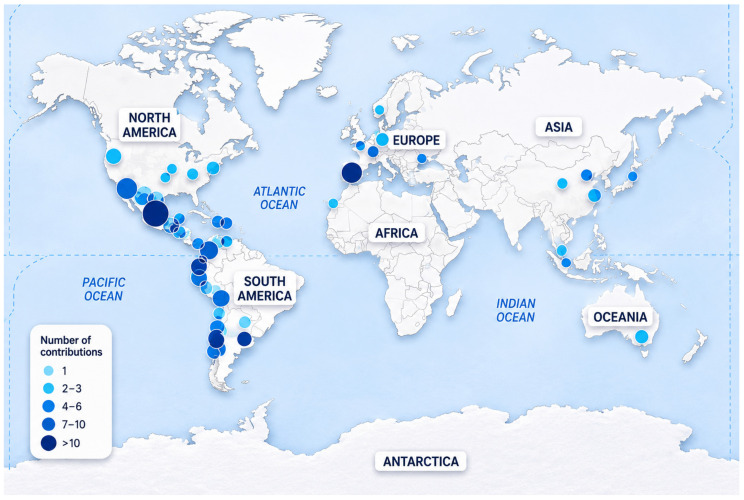
Global distribution of users accessing Panamacani content. The map illustrates the international reach of the journal, with user access distributed across Latin America, Europe, North America, Asia, and Oceania. Blue circles indicate the geographic location of users, and circle size reflects the number of contributions according to the scale shown in the legend. Dashed blue lines represent graphical delimiters used to frame the global map and do not correspond to geographic, political, or analytical boundaries.

In addition to thematic diversification, the journal demonstrates international reach beyond its institutional context. User access has been recorded in more than 20 countries across Latin America, Europe, North America and Asia, including Argentina, Spain, the United States, China and Italy (Figure 5). This geographic distribution highlights the capacity of the initiative to extend beyond local academic boundaries and engage with a broader international audience.

## 4. Discussion

The present study provides a practice-based analysis of the *Panamacani* initiative, illustrating how an editorial-based science communication initiative can be implemented within a higher education setting and how its participation patterns, institutional reach, and audience visibility evolved over time. The findings demonstrate a progressive transition from a local pedagogical activity to a more complex and outward-facing communication platform, supported by both structural and engagement-related indicators.

One of the most relevant findings is the diversification of institutional participation. While the Universidad de Colima remains the primary contributor (70.7%), the presence of external institutions (29.3%) and their increasing participation across issues, reaching parity in Issue 4, indicates growing recognition beyond the original academic context. This expansion reflects a shift from a localized educational exercise toward a broader collaborative network, a key feature of sustainable science communication initiatives [44,45].

In parallel, the evolution of authorship profiles reveals the consolidation of a hybrid ecosystem. Although undergraduate students still represent a substantial proportion of contributors (44.8%), their participation decreased over time (from 88.9% in Issue 1 to 20.0% in Issue 5), while postgraduate contributions increased markedly (from 0% to 66.7%). This shift suggests not a displacement but an enrichment of the publication environment, where early-career authors coexist with more experienced contributors. Such coexistence may contribute to a more diverse publication environment, although these outcomes were not directly evaluated in the present study [46,47].

The temporal patterns observed in institutional participation and authorship diversification are indicative of a maturation process. The transition from internally driven undergraduate contributions toward a more heterogeneous authorship structure is consistent with the consolidation of editorial processes and a broader academic reach of the journal [48,49,50]. Importantly, the available data suggest that this evolution has occurred while maintaining the initiative’s educational orientation, although the formative impact was not directly assessed [48,51,52].

Despite these advantages, this trend also introduces a potential risk. The increasing participation of postgraduate students represents a critical inflection point for *Panamacani*. While this trend reflects greater visibility and academic reach, it also poses a risk of diluting its original pedagogical mission focused on undergraduate training [53,54]. Thus, this evolution is not inherently positive or negative but depends on the strategic objectives of the editorial committee. If the initiative aims to remain educational, it is necessary to establish clear editorial policies, such as prioritizing undergraduate-led submissions or incorporating mentorship-based review processes. Alternatively, this trajectory may position *Panamacani* as a model of how pedagogically oriented journals can evolve into broader science communication platforms. Future assessments should also incorporate evidence of educational impact, including whether publications represent first authorship experiences and how authors perceive skill development. Additionally, the potential role of the journal as a training platform for editorial teams warrants further exploration, as observed in similar initiatives in other academic contexts [53,54,55,56]. Overall, *Panamacani* illustrates the challenges of balancing training objectives with academic production, highlighting the importance of clearly defining its future direction.

Another key contribution of this study lies in the relationship between thematic focus and audience engagement. Descriptive analysis showed that applied health-related content accounted for the highest proportion of views (54.9% within the categorized subset), with leading articles reaching up to 153 views. In contrast, basic sciences and science and society topics exhibit comparatively lower levels of interaction. These findings indicate that applied health-related content (nutrition and disease management) generated greater visibility within the available platform metrics. However, views alone do not allow direct conclusions regarding audience preferences, knowledge acquisition, or communication effectiveness. This pattern aligns with broader evidence indicating that utility and contextual relevance are central drivers of public engagement with science [7,57].

From an educational perspective, the initiative suggests that editorial processes may function as pedagogical tools that facilitate student participation in scientific communication activities. Participation in writing, peer review and publication exposes students to practices typically encountered at advanced stages of academic training. These activities may provide opportunities to practice skills related to synthesis, critical evaluation and audience-oriented communication; however, the present study did not directly assess competency development [46,58].

The participation of postgraduate students also warrants consideration. Although master’s and doctoral students are expected to develop advanced research competencies during their academic training, opportunities to engage directly with scholarly publishing processes may vary substantially across institutions, programs, and stages of academic development [59]. Activities such as manuscript preparation, adaptation to journal requirements, peer-review interactions, editorial revision, and science dissemination represent important components of academic communication that are not always formally incorporated into postgraduate curricula [24,25,59]. Consequently, participation in an editorial-based initiative may provide valuable experiential learning opportunities related to scientific communication and publication processes, even among contributors with previous research experience [12,26].

The *Panamacani* model also underscores the importance of institutional support in ensuring sustainability. The integration of structured editorial workflows, double-blind peer review and digital platforms enables quality control, continuity and scalability. Without such infrastructure, similar initiatives often remain limited in scope and duration. Thus, the success of this model depends not only on pedagogical design but also on institutional commitment.

Beyond its local context, this study suggests that editorial-based approaches constitute a transferable framework for science communication in higher education. The integration of student participation, peer review, digital dissemination and public engagement activities creates a flexible and scalable model that can be adapted across disciplines and institutional settings. This is particularly relevant in Latin America, where strengthening science communication is essential for improving public understanding of health and scientific issues [60,61].

Several limitations should be acknowledged. First, this study describes a single institutional initiative, which may limit the generalizability of the findings. Second, audience engagement was assessed using article views, a metric that reflects content visibility but does not distinguish unique users, reading depth, interaction quality, or automated traffic. Consequently, engagement and international reach should be interpreted as indicators of visibility rather than direct measures of impact, readership, or knowledge transfer [62]. Third, the study did not include direct assessments of educational outcomes; therefore, it cannot determine whether participation in the editorial process improved communication competencies, critical thinking, scientific writing skills, or other learning outcomes. Finally, many of the indicators analyzed, including authorship patterns, institutional participation, and article views, are generated by the editorial system itself and cannot independently demonstrate educational effectiveness [63,64]. Accordingly, the findings should be interpreted as descriptive indicators of editorial development, participation, and visibility. Future studies should incorporate competency-based assessments, participant surveys, qualitative interviews, longitudinal follow-up, article downloads, and alternative engagement metrics to more comprehensively evaluate both the educational and communication impact of the initiative. Finally, because thematic classification was performed through editorial consensus rather than independent blinded assessment, some degree of subjectivity cannot be completely excluded.

Despite these limitations, the findings provide descriptive evidence regarding the feasibility of integrating science communication practices into disciplinary education. More importantly, they demonstrate that such integration can generate outputs that extend beyond the classroom and contribute to broader communication ecosystems. In this sense, *Panamacani* exemplifies how higher education institutions can move beyond knowledge production to actively participate in its societal translation.

## 5. Conclusions

The present study provides a descriptive analysis of the *Panamacani* initiative, documenting its evolution, participation patterns, institutional expansion, and audience engagement over its first five issues. The findings suggest that editorial-based models may represent a promising approach for integrating science communication activities into higher education, although direct evidence of educational outcomes remains to be established through future evaluation studies. The results describe a transition from a local pedagogical activity to a more diversified platform, characterized by increased external participation and a heterogeneous authorship profile. In parallel, audience engagement patterns indicate that applied health-related content achieved greater visibility, highlighting the importance of relevance in science communication. Overall, *Panamacani* appears to function as both a communication platform and an educational initiative that may provide opportunities for participation in scientific communication activities and public dissemination. This model may provide a useful example of how science communication activities can be incorporated into pharmaceutical and health sciences education, particularly in Latin America. Future studies incorporating competency-based assessments, participant surveys, qualitative feedback, and longitudinal evaluations will be necessary to empirically evaluate the educational impact of this model.

## Figures and Tables

**Table 1:** Distribution of authorship by academic level across issues.

Issue	Undergraduate (*n*, %)	Postgraduate (*n*, %)	Researcher (*n*, %)	Total
1	8 (88.9%)	0 (0.0%)	1 (11.1%)	9
2	4 (36.4%)	1 (9.1%)	6 (54.5%)	11
3	8 (72.7%)	0 (0.0%)	3 (27.3%)	11
4	3 (25.0%)	5 (41.7%)	4 (33.3%)	12
5	3 (20.0%)	10 (66.7%)	2 (13.3%)	15

Undergraduate authors correspond to bachelor-level students; postgraduate authors include master’s and doctoral students; researchers refer to faculty members or professionals affiliated with academic or research institutions. Issue-level counts represent the number of published contributions per issue, classified according to the academic level of the first author. Counts represent the number of published contributions per issue.

**Table 2:** Distribution of contributions by institutional affiliation and authorship type across issues.

Issue	Internal (*n*)	External (*n*)	Undergraduate (*n*)	Postgraduate (*n*)	Researcher (*n*)
1	8	1	8	0	1
2	7	4	4	1	6
3	9	2	8	0	3
4	6	6	3	5	4
5	12	3	3	10	2

Authorship classification (undergraduate, postgraduate, researcher) was based on the academic level of the first author, considered the primary contributor. Counts represent the number of published contributions per issue.

## Data Availability

The original contributions presented in this study are included in the article; further inquiries can be directed to the corresponding author.
